# Acceptance and commitment therapy combined with usual care improves psychosocial outcomes and reduces complications in patients with permanent colostomies after colorectal cancer surgery: a retrospective cohort study

**DOI:** 10.3389/fsurg.2025.1693290

**Published:** 2025-10-24

**Authors:** Yali Shi, Hongwei Yu, Lihui Wang, Haiqiang Zhang

**Affiliations:** 1 The Second Hospital of Hebei Medical University, Infection Control Office, Shijiazhuang, China; 2 Nursing Department, The Second Hospital of Hebei Medical University, Shijiazhuang, China; 3 Medical Imaging Department, The Second Hospital of Hebei Medical University, Shijiazhuang, China; 4 Gastrointestinal Surgery, Hernia and Abdominal Wall Surgery, The Second Hospital of Hebei Medical University, Shijiazhuang, China

**Keywords:** acceptance and commitment therapy (act), permanent colostomy, stoma-relatedcomplications, quality of life, colorectal cancer surgery

## Abstract

**Background:**

Permanent colostomies after colorectal cancer surgery can seriously affect patients' quality of life (QoL) and psychosocial adjustment. Research on the benefits of Acceptance and Commitment Therapy (ACT) for this group is still limited.

**Objective:**

To examine whether adding ACT to standard stoma care improves self-efficacy, resilience, QoL, and stoma-related complication rates compared with standard care alone.

**Methods:**

This single-center retrospective cohort study (2022–2024) included 120 patients with permanent colostomies. After 1:1 propensity score matching (60 patients per group, caliper = 0.2 SD), one group received an 8-session ACT program over 6 weeks alongside usual care, while the control group received usual care only. Outcomes were measured at 3 months (T1) and 6 months (T2) post-surgery. Primary outcomes were self-efficacy (C-SSES), resilience (CD-RISC), and stoma-related QoL (Stoma-QOL). Secondary outcomes included stoma complications. Linear mixed-effects models and conditional logistic regression were applied for analysis.

**Results:**

Significant improvements over time were observed in the ACT group compared with controls (all *p* < 0.001). At T2, the ACT group showed higher self-efficacy [mean difference (MD) = 14.7, 95% CI: 10.9–18.5; *d* = 0.92], resilience (MD = 11.1, 95% CI: 7.8–14.4; d = 0.89), and QoL (MD = 12.3, 95% CI: 8.7–15.9; *d* = 0.86). ACT also reduced overall complication rates (33.3% vs. 51.7%; OR = 0.48, 95% CI: 0.24–0.96), particularly dermatitis (16.7% vs. 31.7%; OR = 0.43, 95% CI: 0.19–0.99). At T2, self-efficacy, resilience, and QoL were strongly correlated (all *r* ≥ 0.65, *p* < 0.001).

**Conclusion:**

Adding ACT to routine stoma care improves self-efficacy, resilience, and quality of life, while also lowering complication rates in patients with permanent colostomies. These findings suggest ACT is a valuable supportive therapy in stoma care.

## 1 Introduction

Colorectal cancer (CRC) remains a leading global health challenge, ranking third in incidence and second in mortality worldwide. According to the 2021 Global Burden of Disease (GBD) study, CRC caused approximately 2.19 million new cases and 1.09 million deaths globally, with the age-standardized incidence rate continuing to rise in most regions, despite modest decreases in mortality and disability-adjusted life years during the same period (1, 2). Major lifestyle-related factors, including high red meat consumption, obesity, physical inactivity, and elevated fasting glucose are estimated to account for a substantial portion of this burden (2, 3).

Surgical resection remains the cornerstone of CRC treatment, often offering curative potential. However, a significant number of patients require stoma formation. Registry-based evidence from Sweden suggests that between 18% and 25% of patients undergoing anterior resection for rectal cancer have stomas that remain unreversed at two years postoperatively (4). In cases of emergency or obstructive CRC, approximately 20% of patients receive a permanent stoma (5). Older age, higher carcinoembryonic antigen (CEA) levels, and emergency surgery significantly predict permanent stoma formation (5, 6).

Permanent stoma creation profoundly affects patient quality of life and long-term adaptation. A study showed that over 80% of long-term ostomy patients experienced stoma-related difficulties, including skin irritation, leakage, parastomal hernia, and body image concerns (7). Similarly, qualitative research in Chinese CRC survivors highlighted daily life disruption and psychosocial distress even years after surgery (8). Additionally, only about 30% of patients return to work following permanent stoma surgery at a median of 6 months, with complications and lack of support contributing significantly to this low rate (9).

Current research on psychological interventions for permanent colostomy patients has significant gaps. Most prior studies focus on temporary stomas or general cancer distress, overlooking the unique, lifelong psychosocial challenges of irreversible ostomies. Acceptance and Commitment Therapy (ACT), as a prominent “third wave” cognitive behavioral intervention, emphasizes acceptance of present-moment experience and value-based behavior change, and has shown promising effects in chronic illness, anxiety, and depression populations (10, 11). Crucially, evidence is lacking regarding the impact of structured therapies like ACT specifically on core adaptation mechanisms, self-efficacy, resilience, and stoma-specific quality of life. Furthermore, few studies integrate psychological interventions with standard stoma care protocols or examine their potential influence on clinical outcomes such as complication rates. These limitations hinder the development of targeted support strategies for this vulnerable population.

This study aimed to evaluate whether adding ACT to usual stoma care improves key psychosocial and clinical outcomes in colorectal cancer patients with permanent colostomies compared to usual care alone. Specifically, we assessed group differences in self-efficacy, psychological resilience, and stoma-related quality of life at 3 and 6 months postoperatively. Secondary objectives included comparing stoma complication rates and exploring correlations between psychosocial outcomes to understand potential therapeutic mechanisms.

### 2.1 Study design

This single-center retrospective cohort study evaluated the effects of ACT combined with usual care vs. usual care alone on self-efficacy, resilience, and quality of life (QoL) in patients with permanent colostomies following colorectal cancer surgery. Data from patients who underwent surgery between January 1, 2022, and December 31, 2024, were extracted from electronic medical records (EMR) and institutional psychological assessment databases. The study protocol was approved by the Institutional Review Board of The Second Hospital of Hebei Medical University, with a waiver of informed consent granted due to the anonymous retrospective use of routinely collected clinical data. Reporting adhered to the Strengthening the Reporting of Observational Studies in Epidemiology (STROBE) guidelines. All data were de-identified and managed under strict confidentiality protocols in compliance with the Declaration of Helsinki.

### 2.2 Study participants

#### 2.2.1 Inclusion criteria

Patients were eligible if they met all of the following:

1. Age ≥18 years;
2. Histopathologically confirmed colorectal adenocarcinoma per WHO diagnostic criteria (12);
3. Underwent curative-intent surgery with creation of a permanent end colostomy (defined as non-reversible stoma without planned restoration of bowel continuity) (13);
4. Minimum postoperative survival ≥12 months to ensure capture of all follow-up endpoints;
5. Initiation of institutional standard stoma care within 30 days postoperatively;
6. Completion of validated outcome measures (self-efficacy, resilience, QoL) at both 3-month (T1) and 6-month (T2) follow-ups.

#### 2.2.2 Exclusion criteria

Patients were excluded for any of the following:

1. Pre-existing severe cognitive impairment (Mini-Mental State Examination score <18) (14) or major psychiatric disorders (e.g., schizophrenia, bipolar disorder) per DSM−5 criteria (15);
2. 30% missing data in medical records or outcome questionnaires;
3. History of prior stoma creation or conversion from temporary to permanent stoma if initial surgery occurred before January 1, 2022;
4. Concurrent enrollment in interventional trials involving psychological or stoma-management therapies;
5. Emergency surgery or palliative stoma creation.

#### 2.2.3 Sample size justification

*a priori* power analysis was conducted using G*Power 3.1 based on prior ACT trials in cancer populations (10). Targeting a moderate effect size (Cohen's *d* = 0.6) for QoL improvement with *α* = 0.05% and 90% power, 64 patients per group were required. Accounting for 20% attrition from incomplete follow-up data, 154 patients (77 per group) were targeted. Final analytic samples are reported in Section 3.1.

### 2.3 Group assignment and interventions

#### 2.3.1 ACT + Usual care group

Patients allocated to the ACT + Usual Care group received a structured 6-week Acceptance and Commitment Therapy (ACT) protocol adapted from Hayes et al.'s core manual (16), delivered adjunctively to standard stoma care. The intervention comprised eight 90-minute sessions (two sessions weekly for Weeks 1–4, followed by biweekly sessions for Weeks 5–6), administered in a hybrid format: four group sessions (6–8 patients/group) focused on psychoeducation and experiential exercises, supplemented by four individual sessions for personalized goal implementation. Licensed clinical psychologists with ≥2 years of ACT specialization facilitated all sessions, adhering to a predefined curriculum:

1. Weeks 1–2: Acceptance and Cognitive Defusion—Techniques included “leaves on a stream” (defusion from stoma-related distress) and “physicalizing” discomfort to reduce avoidance behaviors.
2. Weeks 3–4: Present-Moment Awareness and Self-as-Context—Mindfulness training (e.g., “stoma-scan meditation”) and perspective-taking exercises to decouple self-identity from stoma-related shame.
3. Weeks 5–6: Values Clarification and Committed Action—Identification of post-cancer life values (e.g., family engagement, social reintegration) and behavior activation plans (e.g., incremental social outings with stoma management strategies).

Treatment fidelity was monitored via session checklists documenting adherence to ACT processes (≥80% protocol compliance required). Concurrently, all patients received identical standard stoma care: daily peristomal skin assessments by enterostomal therapists, customized ostomy appliance fitting, dietary optimization (low-residue protocols), and complication management per.

#### 2.3.2 Usual care group

Patients in the Usual Care group received exclusively the standard stoma care protocol described above, without structured psychological interventions. Routine nursing support included brief (<15 min) emotional reassurance during stoma education sessions but excluded any ACT-consistent techniques (e.g., values work, defusion exercises) or formal psychotherapy referrals. Psychological care was limited to crisis management for acute distress (e.g., suicidal ideation), documented in <5% of cases.

### 2.4 Study variables and measurement tools

#### 2.4.1 Primary outcomes

Self-efficacy was assessed using the validated Chinese version of the Stoma Self-Efficacy Scale (C-SSES) (17). This 15-item instrument measures three domains: stoma care (5 items), social engagement (6 items), and emotional management (4 items). Responses are recorded on a 10-point Likert scale (1 = “not at all confident” to 10 = “extremely confident”), with total scores ranging from 15 to 150. Higher scores indicate greater self-efficacy. The scale demonstrates excellent internal consistency (Cronbach's *α* = 0.92) and test-retest reliability (ICC = 0.87) in ostomy populations (17). Assessments occurred at postoperative 3 months (T1) and 6 months (T2).

Psychological resilience was evaluated with the 25-item Chinese Connor-Davidson Resilience Scale (CD-RISC) (18). This tool captures five dimensions: personal competence (8 items), trust/tolerance (7 items), positive acceptance (5 items), control (3 items), and spiritual influences (2 items). Items are rated on a 5-point scale (0 = “never true” to 4 = “always true”), yielding total scores from 0 to 100. The Chinese version shows strong psychometric properties (*α* = 0.91; convergent validity *r* = 0.78 with GSE) (18), with measurements at T1 and T2.

Quality of Life (QoL) was measured using the Stoma-QOL questionnaire (19), a disease-specific instrument validated for Chinese colorectal ostomy patients. Its 20 items span four subscales: physical concerns (6 items), psychological well-being (5 items), social function (4 items), and stoma-specific issues (5 items). Responses use a 4-point Likert frequency scale (“always” to “never”), linearly transformed to 0–100 scales where higher scores reflect better QoL. The Stoma-QOL has demonstrated high reliability (*α* = 0.86–0.94 across subscales) and discriminant validity in surgical cohorts (19), assessed at T1 and T2.

#### 2.4.2 Secondary outcomes

Stoma-related complications were extracted from surgical follow-up records using predefined criteria: dermatitis [Peristomal Skin Assessment Guide ≥ Grade 2 (20)], stenosis (inability to pass a 12-mm endoscope), prolapse (>2 cm descent beyond skin), and parastomal hernia [clinical/CT-confirmed EHS classification (21)].

#### 2.4.3 Covariates

Demographic covariates included age, sex, education level (categorized: ≤middle school, high school, ≥college), marital status, residence (urban/rural), employment status, monthly household income per capita (RMB), and primary caregiver relationship. Clinical covariates comprised TNM stage [AJCC 8th edition (22)], surgical approach (laparoscopic vs. open), stoma type (sigmoid vs. transverse), postoperative complications [Clavien-Dindo grade (23)], adjuvant therapy, and comorbidities [Charlson Comorbidity Index (24)]. Baseline outcome scores (self-efficacy, resilience, QoL) at postoperative 1 month (T0) were included to model longitudinal changes.

### 2.5 Data collection and management

Data extraction was performed by two trained research associates blinded to group allocation and study hypotheses, using a standardized electronic case report form (eCRF) developed in REDCap (Research Electronic Data Capture, Vanderbilt University). All source data, including demographic/clinical variables from EMR, intervention details from psychology service logs, and outcome measures from the institutional assessment database—underwent independent dual entry with automated discrepancy flagging. Discrepancies were resolved through consensus or third-party adjudication by the principal investigator. Following extraction, data underwent comprehensive cleaning: (1) range checks for numerical variables (e.g., QoL scores 0–100), (2) consistency validation (e.g., surgery dates preceding follow-ups), and (3) logic verification (e.g., exclusion of deceased patients at follow-up timepoints). De-identified datasets were stored on a password-encrypted hospital server with role-based access controls, compliant with ISO 27001 data security standards. Missing data patterns were documented quantitatively; primary analyses utilized complete-case analysis (CCA), while sensitivity analyses employed multiple imputation (MI) using chained equations (MICE algorithm, 20 imputed datasets) under the missing-at-random (MAR) assumption, incorporating auxiliary variables (e.g., baseline distress scores) to strengthen imputation models.

### 2.6 Statistical analysis

All analyses were performed using R software (version 4.3.1) with the MatchIt, lme4, and mice packages. Descriptive statistics presented continuous variables as mean ± standard deviation for normally distributed data (Shapiro–Wilk test *p* > 0.05) or median (interquartile range) for non-normal distributions, while categorical variables were summarized as frequencies (percentages). Pre-match baseline comparisons employed independent *t*-tests (normal distributions with equal variance per Levene's test) or Mann–Whitney U tests for continuous variables, and chi-square or Fisher's exact tests for categorical variables. Primary analyses utilized propensity score matching (PSM) to address confounding: all baseline covariates (demographics, clinical characteristics, and T0 outcome scores) were included in a logistic regression model to estimate propensity scores. 1:1 nearest-neighbor matching with a caliper width of 0.2 SD ensured balance, assessed via standardized mean differences (SMD < 0.10 considered balanced). Post-match, linear mixed models (LMM) analyzed longitudinal outcomes (self-efficacy, resilience, QoL) with fixed effects for group (ACT + Usual Care vs. Usual Care), time (T1/T2), and group-by-time interaction, plus random intercepts for subjects. Significant interactions (*p* < 0.05) prompted stratified timepoint analyses, while nonsignificant interactions interpreted main group effects. Dichotomous secondary outcomes (e.g., complications) underwent conditional logistic regression. To contextualize clinical significance in the absence of established minimal clinically important differences (MCIDs), effect sizes were calculated using Cohen's d (adjusted mean difference divided by pooled baseline standard deviation). Effect sizes were interpreted using conventional thresholds: small (*d* = 0.20), medium (*d* = 0.50), and large (*d* ≥ 0.80) (25). Additionally, improvements exceeding 0.5 times the baseline standard deviation (0.5 × SD) were considered clinically relevant based on distribution-based methods (26). These approaches provide standardized metrics for evaluating intervention impact when disease-specific MCIDs are unavailable. Sensitivity analyses included: (1) multivariable-adjusted LMM in the full cohort including all covariates, and (2) complete-case vs. multiple imputation comparisons. Correlation analyses used Pearson (normally distributed) or Spearman (non-normal) coefficients to evaluate associations between self-efficacy, resilience, and QoL at each timepoint. Statistical significance was defined as two-tailed *p* < 0.05, with Bonferroni correction for multiple comparisons (e.g., QoL subscales). All models reported effect sizes (mean differences [MD], odds ratios [OR], regression coefficients [β], correlation coefficients [*r*/*ρ*]) with 95% confidence intervals.

## 3 Results

### 3.1 Patient selection and baseline characteristics

**Figure 1:**
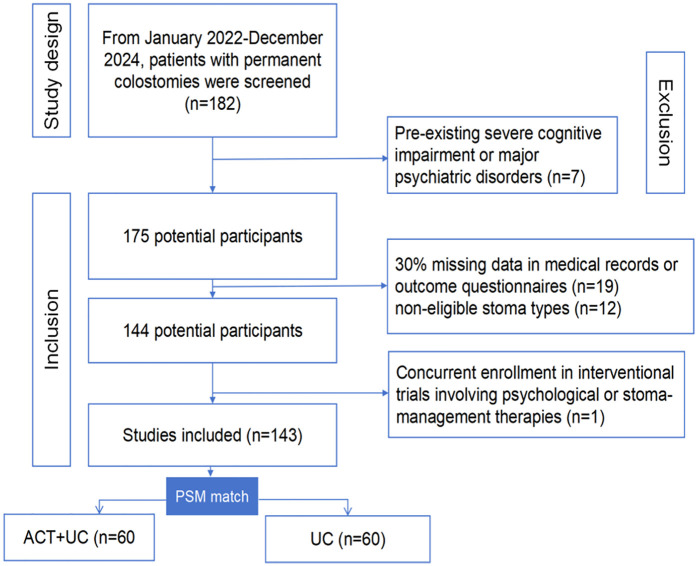
Inclusion and exclusion flowchart.

During the study period (January 2022–December 2024), 182 patients with permanent colostomies were screened. After excluding 39 ineligible patients (cognitive impairment: *n* = 7; > 30% missing data: *n* = 19; concurrent trials: *n* = 1; non-eligible stoma types: *n* = 12), 143 patients comprised the full cohort (Figure 1). Propensity score matching (1:1 caliper = 0.2 SD) successfully balanced 60 patient pairs (ACT + Usual Care: *n* = 60; Usual Care: *n* = 60), with baseline characteristics detailed in Table 1. After matching, all standardized mean differences fell below 0.1 (range: 0.00–0.08), confirming successful balance across demographic, clinical, and psychological variables for subsequent outcome analyses.

**Table 1:** Baseline characteristics before and after propensity score matching.

Characteristic	Before matching				After matching			
	ACT + UC (*n* = 68)	UC (*n* = 75)	*p*-value	SMD	ACT + UC (*n* = 60)	UC (*n* = 60)	*p*-value	SMD
Demographics								
Age, years	58.1 ± 8.9	62.4 ± 9.3	0.008	0.47	59.0 ± 8.7	59.3 ± 8.9	0.85	0.03
Female sex	30 (44.1%)	36 (48.0%)	0.64	0.08	27 (45.0%)	26 (43.3%)	0.86	0.03
College education	38 (55.9%)	28 (37.3%)	0.03	0.38	32 (53.3%)	31 (51.7%)	0.86	0.03
Urban residence	49 (72.1%)	46 (61.3%)	0.18	0.23	43 (71.7%)	42 (70.0%)	0.84	0.04
Income >¥10,000/month	41 (60.3%)	33 (44.0%)	0.05	0.33	36 (60.0%)	35 (58.3%)	0.86	0.03
Clinical Features								
TNM Stage III/IV	42 (61.8%)	46 (61.3%)	0.96	0.01	37 (61.7%)	36 (60.0%)	0.86	0.03
Laparoscopic surgery	52 (76.5%)	54 (72.0%)	0.55	0.10	46 (76.7%)	45 (75.0%)	0.83	0.04
Sigmoid colostomy	58 (85.3%)	64 (85.3%)	1.00	0.00	51 (85.0%)	51 (85.0%)	1.00	0.00
Clavien-Dindo ≥ II	18 (26.5%)	22 (29.3%)	0.71	0.06	16 (26.7%)	17 (28.3%)	0.84	0.04
Adjuvant chemotherapy	54 (79.4%)	58 (77.3%)	0.76	0.05	48 (80.0%)	47 (78.3%)	0.83	0.04
Charlson Index ≥2	21 (30.9%)	27 (36.0%)	0.53	0.11	19 (31.7%)	18 (30.0%)	0.85	0.04
Hypertension	25 (36.8%)	29 (38.7%)	0.82	0.04	22 (36.7%)	23 (38.3%)	0.86	0.03
Diabetes mellitus	14 (20.6%)	18 (24.0%)	0.64	0.08	12 (20.0%)	13 (21.7%)	0.83	0.04
Current smoker	19 (27.9%)	25 (33.3%)	0.50	0.12	17 (28.3%)	18 (30.0%)	0.84	0.04
Baseline Scores (1-month)								
Self-efficacy (SSES)	100.3 ± 16.2	110.5 ± 15.8	<0.001	0.64	102.1 ± 15.6	103.3 ± 15.2	0.67	0.08
Resilience (CD-RISC)	59.8 ± 11.4	66.2 ± 10.9	<0.001	0.57	61.0 ± 10.8	61.7 ± 10.5	0.71	0.07
Quality of Life (Stoma-QOL)	53.7 ± 9.8	60.5 ± 9.2	<0.001	0.71	55.2 ± 9.4	55.9 ± 9.0	0.68	0.08

Data: mean ± SD or *n* (%).

ACT + UC, ACT + Usual Care; UC, usual care; SMD, standardized mean difference.

### 3.2 Comparison of primary outcomes using linear mixed models

Linear mixed models revealed significant group-by-time interactions for all primary outcomes (all *p* < 0.001), indicating superior improvements from 3 to 6 months postoperatively in the ACT + Usual Care group compared to Usual Care alone. For self-efficacy, ACT + Usual Care patients demonstrated a 15.8-point increase from T1 to T2 vs. 4.2-points in controls (net additional gain: 11.6 points). Similarly, resilience improved by 13.4 points in the intervention group compared to 5.1 points in controls, while quality of life showed a 14.7-point vs. 5.3-point differential improvement. Time effects were universally significant (all *p* < 0.001), confirming overall score progression. Group main effects were non-significant for self-efficacy and resilience (*p* > 0.05) but significant for quality of life (*β* = 4.1, *p* = 0.016), reflecting ACT's holistic impact. All models showed excellent fit (AIC/BIC reduction >15% vs. null models) (Table 2).

**Table 2:** Fixed effects from linear mixed models for primary outcomes.

Outcome and effect	*β* (95% CI)	*p*-value	Model fit (AIC/BIC)
Self-efficacy (SSES)			1,245.3/1,268.9
Group (ACT + UC vs. UC)	2.3 (−0.8, 5.4)	0.147	
Time (T2 vs. T1)	9.7 (7.1, 12.3)	<0.001	
Group × Time interaction	11.6 (8.2, 15.0)	<0.001	
Resilience (CD-RISC)			982.4/1,006.1
Group (ACT + UC vs. UC)	1.6 (−1.2, 4.4)	0.261	
Time (T2 vs. T1)	8.9 (6.6, 11.2)	<0.001	
Group × Time interaction	8.3 (5.3, 11.3)	<0.001	
Quality of Life (Stoma-QOL)			1,087.6/1,111.3
Group (ACT + UC vs. UC)	4.1 (0.8, 7.4)	0.016	
Time (T2 vs. T1)	9.8 (7.3, 12.3)	<0.001	
Group × Time interaction	9.4 (6.0, 12.8)	<0.001	

Models adjusted for baseline scores (T0) with random intercepts for subjects.

ACT + UC, ACT + Usual Care; UC, Usual Care; β, regression coefficient; CI, confidence interval.

### 3.3 Comparison of primary outcomes using multiple linear regression

**Figure 2:**
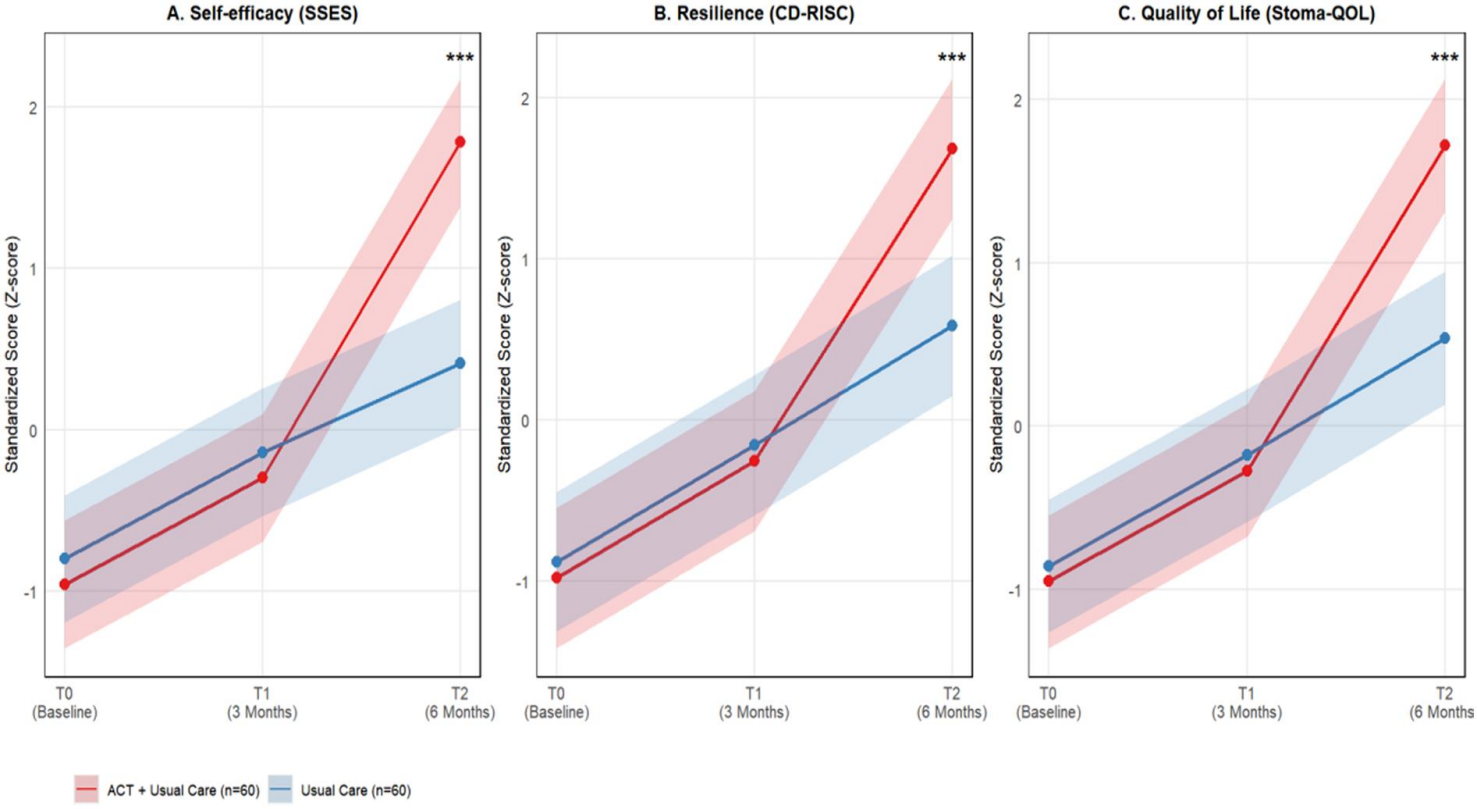
Trajectories of primary outcomes from baseline to 6 months post-surgery. The figure illustrates standardized scores (*z*-scores) for **(A)** self-efficacy (SSES), **(B)** resilience (CD-RISC), and **(C)** stoma-specific quality of life (Stoma-QOL) across three time points: baseline (T0), 3 months (T1), and 6 months (T2) post-surgery. Red lines represent the ACT plus usual care intervention group (*n* = 60), while blue lines represent the usual care control group (*n* = 60). Shaded areas indicate 95% confidence intervals. ***p < 0.001) for all three outcomes.

Adjusted multivariate regression analyses at specific timepoints confirmed the progressive benefits of ACT + Usual Care. At 3 months postoperatively (T1), no significant between-group differences were observed across outcomes after adjusting for baseline scores and clinical covariates. The magnitude of improvement in the ACT + Usual Care group at T2 represented large effect sizes (Cohen's *d* = 0.86–0.92), indicating substantial clinical relevance (Table 3, Figure 2). These gains represented 86%–92% of the baseline standard deviation, exceeding the 0.5 SD benchmark for clinical importance.

**Table 3:** Adjusted mean differences and effect sizes at follow-up timepoints.

Outcome	Timepoint	AMD (95% CI)	*p*-value	Effect size (Cohen's *d*)
Self-efficacy (SSES)	T1 (3-month)	3.1 (−0.4, 6.6)	0.082	0.19
	T2 (6-month)	14.7 (10.9, 18.5)	<0.001	0.92 (Large)
Resilience (CD-RISC)	T1 (3-month)	2.8 (−0.7, 6.3)	0.117	0.25
	T2 (6-month)	11.1 (7.8, 14.4)	<0.001	0.89 (Large)
Quality of Life (Stoma-QOL)	T1 (3-month)	2.9 (−0.6, 6.4)	0.103	0.31
	T2 (6-month)	12.3 (8.7, 15.9)	<0.001	0.86 (Large)

Effect size interpretation: Small (*d* = 0.2), Medium (*d* = 0.5), Large (*d* ≥ 0.8). Models adjusted for baseline scores (T0), age, education, income, TNM stage, and Charlson Comorbidity Index.

ACT + UC, ACT + Usual Care; AMD, adjusted mean difference; CI, confidence interval.

### 3.4 Secondary outcomes

**Figure 3:**
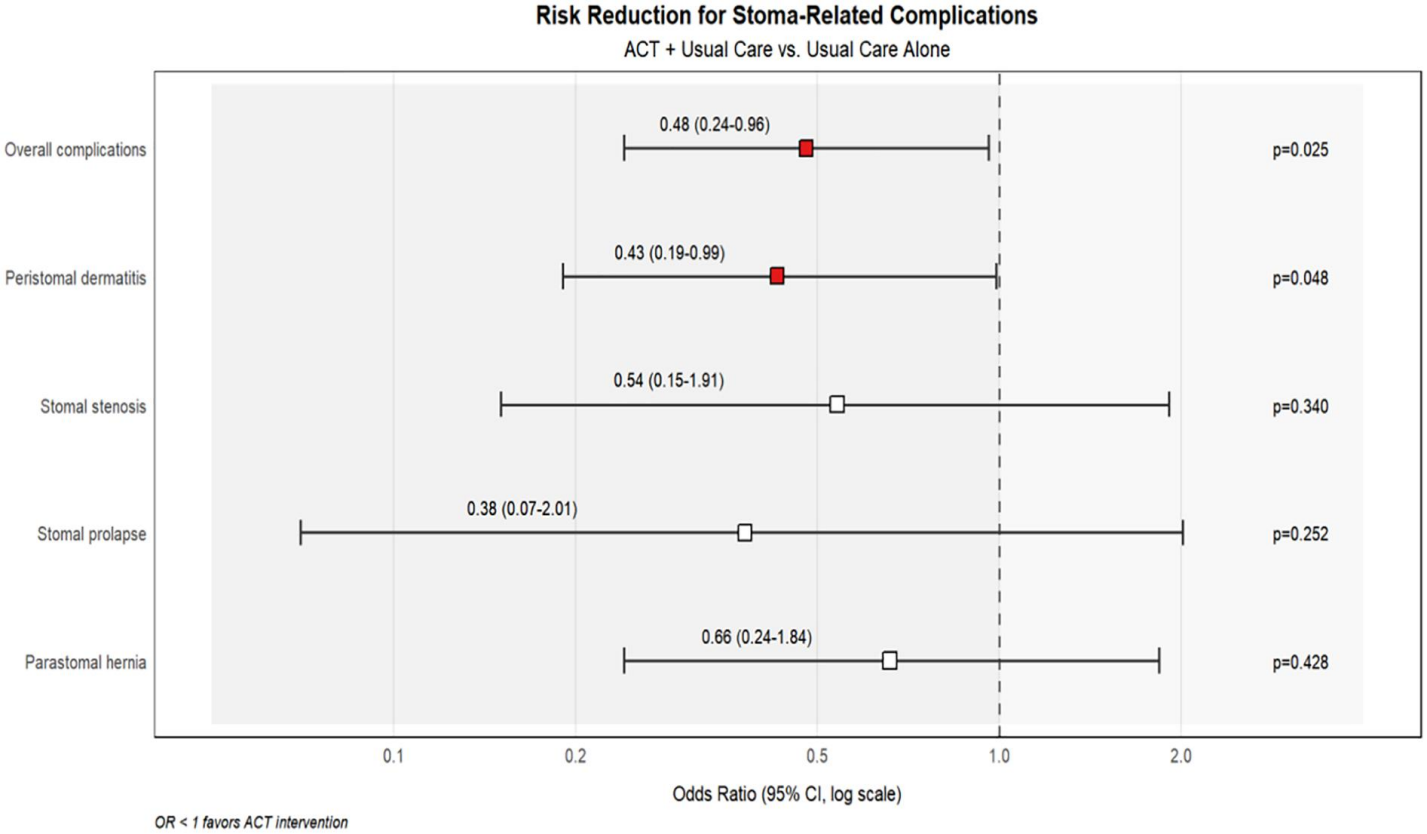
Forest plot showing risk reduction for stoma-related complications with ACT intervention compared to usual care alone. Odds ratios (OR) with 95% confidence intervals are displayed on a logarithmic scale. The blue shaded area (OR < 1) indicates reduced risk in the ACT intervention group, while the light red area (OR > 1) would represent increased risk. Red squares indicate statistically significant reductions (*p* < 0.05), while white squares represent non-significant trends.

Analysis of stoma-related complications revealed clinically important differences between groups, particularly for dermatitis which is closely linked to self-care adherence. The ACT + Usual Care group demonstrated significantly lower overall complication rates compared to Usual Care alone (33.3% vs. 51.7%, *p* = 0.025), with the most pronounced reduction in dermatitis incidence (16.7% vs. 31.7%, *p* = 0.048). Other complications showed non-significant trends favoring the intervention group (Table 4, Figure 3).

**Table 4:** Stoma-related complications within 6 months postoperatively.

Complication type	ACT + UC (*n* = 60)	UC (*n* = 60)	OR (95% CI)	*p*-value
Peristomal dermatitis	10 (16.7%)	19 (31.7%)	0.43 (0.19–0.99)	0.048
Stomal stenosis	4 (6.7%)	7 (11.7%)	0.54 (0.15–1.91)	0.340
Stomal prolapse	2 (3.3%)	5 (8.3%)	0.38 (0.07–2.01)	0.252
Parastomal hernia	7 (11.7%)	10 (16.7%)	0.66 (0.24–1.84)	0.428
Overall complications	20 (33.3%)	31 (51.7%)	0.48 (0.24–0.96)	0.025

ACT + UC, ACT + Usual Care; UC, usual care; OR, odds ratio; CI, confidence interval.

### 3.5 Correlations among self-efficacy, resilience, and quality of life

**Figure 4:**
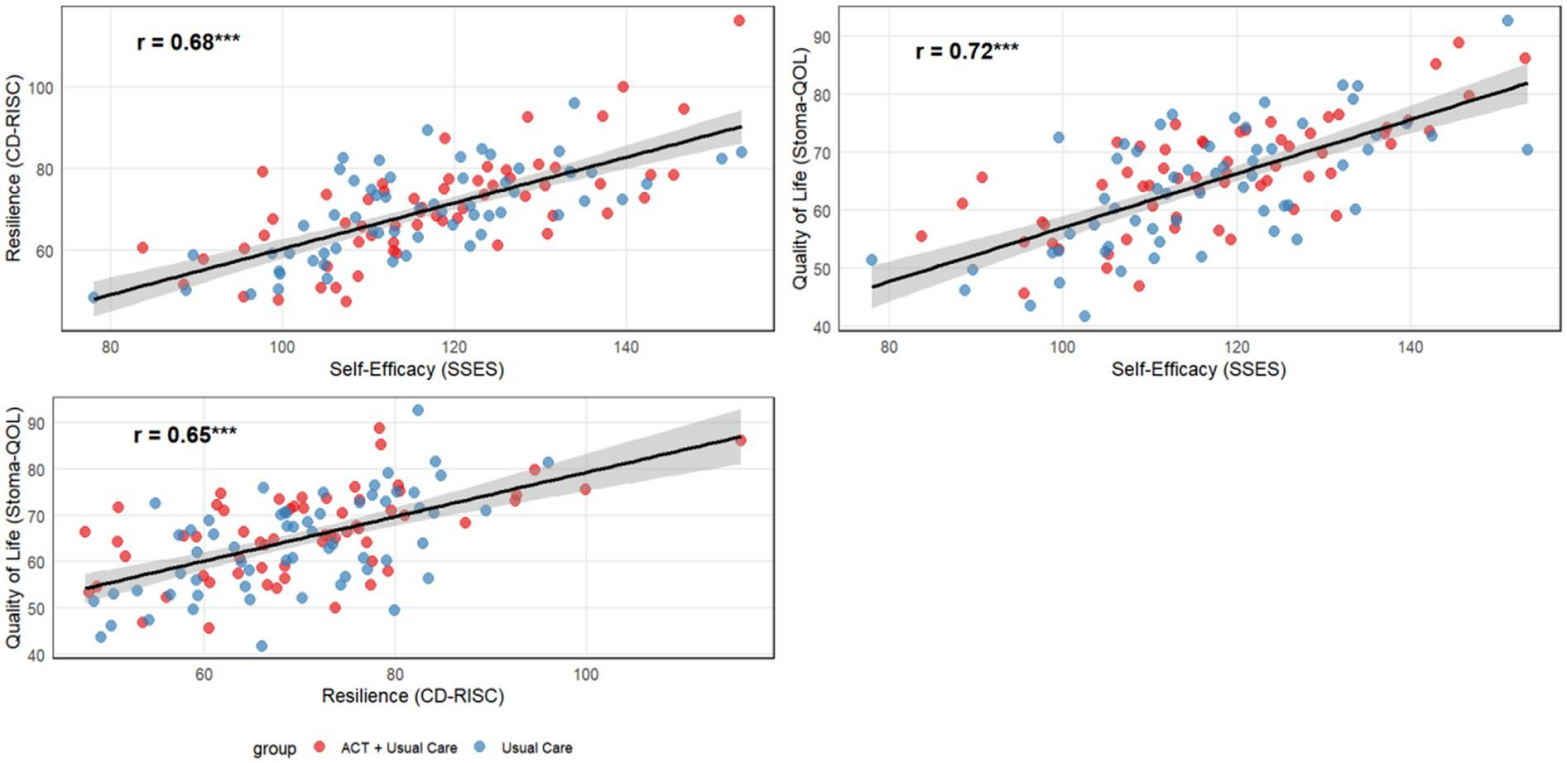
Scatter plots illustrating correlations among primary outcomes at 6-month follow-up (T2). Red points represent ACT intervention participants (*n* = 60) and blue points represent usual care participants (*n* = 60). Strong positive correlations were observed between all three outcomes (all *p* < 0.001): self-efficacy showed the strongest association with quality of life (*r* = 0.72), followed by the relationship between self-efficacy and resilience (*r* = 0.68), and resilience with quality of life (*r* = 0.65). Black trend lines represent the overall correlation across both groups.

At 6 months postoperatively (T2), strong positive correlations were observed between self-efficacy, resilience, and overall quality of life, supporting the hypothesized mechanistic relationships. Self-efficacy demonstrated the strongest association with quality of life (*r* = 0.72), accounting for over 50% of shared variance, while resilience showed substantial correlation with both self-efficacy (*r* = 0.68) and quality of life (*r* = 0.65) (Table 5, Figure 4). All correlations were statistically significant (*p* < 0.001) and exceeded conventional thresholds for large effect sizes.

**Table 5:** Correlation matrix at 6-month follow-up (T2).

Variable	Self-efficacy (SSES)	Resilience (CD-RISC)	Quality of life (Stoma-QOL)
Self-Efficacy (SSES)	1.00	–	–
Resilience (CD-RISC)	0.68***	1.00	–
Quality of Life (Stoma-QOL)	0.72***	0.65***	1.00

*** *p* < 0.001 (two-tailed).

Pearson correlation coefficients reported (*n* = 120).

## 4 Discussion

This retrospective cohort study demonstrated that incorporating ACT into standard postoperative stoma care significantly improved patients' self-efficacy, psychological resilience, and stoma-specific quality of life over a 6-month period following permanent colostomy for colorectal cancer. These findings confirmed both primary hypotheses: (1) that ACT + Usual Care would be associated with greater improvements in self-efficacy, resilience, and quality of life than Usual Care alone, and (2) that these psychosocial constructs would be positively correlated with one another at follow-up. These associations remained robust after adjustment for potential confounders and were supported by large effect sizes, suggesting that ACT may contribute meaningfully to patient recovery trajectories.

The observed enhancement in self-efficacy among ACT recipients was substantial (adjusted mean difference = 14.7 points), far exceeding the 0.5 standard deviation threshold typically interpreted as clinically meaningful. This aligns with ACT's central aim to promote psychological flexibility through techniques such as cognitive defusion and committed action. By facilitating disengagement from maladaptive thoughts, particularly those tied to stoma-related stigma or incompetence, and encouraging values-driven behavior, ACT may empower patients to regain control over their bodily functions and social participation. These mechanisms are supported by prior randomized trials demonstrating ACT's capacity to increase self-efficacy in populations managing chronic somatic diseases, including cancer and inflammatory bowel disease (10, 27). Studies in patients with colorectal cancer stoma have shown that the self-management ability of Chinese colorectal cancer patients is at a moderate level, and the self-efficacy of colorectal cancer stoma patients is affected by social support, which ultimately leads to the change of their self-management ability (28). Furthermore, enhanced self-efficacy may directly impact clinical outcomes; for instance, reduced peristomal dermatitis in the ACT group (16.7% vs. 31.7%) suggests improved stoma care adherence, a likely behavioral correlate of self-efficacy enhancement (29).

The significant gains in psychological resilience (mean difference = 11.1 points) observed in the ACT + Usual Care group are similarly consistent with the therapy's theoretical framework. ACT emphasizes acceptance of aversive internal experiences, present-moment awareness, and perspective-taking—all of which may buffer individuals against stressors associated with life-altering surgeries such as permanent ostomy. Prior studies in cancer populations have shown that ACT interventions yield moderate-to-large improvements in resilience, especially when acceptance strategies are emphasized (30, 31). The effect size observed here (Cohen's d = 0.89) exceeded those reported in earlier work (32), potentially reflecting the specific emotional and existential challenges unique to patients with permanent colostomies. These patients often face irreversible body image alterations and social role disruptions, which may render them particularly amenable to ACT's acceptance- and values-based interventions.

QoL also improved substantially, with a mean increase of 12.3 points in the ACT group. This improvement was not merely statistically significant but also clinically relevant, highlighting ACT's potential to address both psychological and functional domains of wellbeing. The positive correlations observed between QoL, self-efficacy (*r* = 0.72), and resilience (*r* = 0.65) underscore a plausible interdependent mechanism: increased self-confidence in stoma management and enhanced emotional flexibility may synergistically support adaptation, leading to better overall wellbeing. These findings are concordant with prior longitudinal studies identifying self-efficacy and resilience as independent predictors of QoL in ostomy and other oncology populations (33, 34).

Compared with previous studies, the current research extends the evidence base in important ways. While two prior RCTs evaluated ACT in patients with cancer, few have focused specifically on individuals with permanent colostomies (35, 36). Additionally, our study included a larger sample size, implemented rigorous confounding control via propensity score matching, and assessed outcomes at multiple timepoints, thereby enabling analysis of temporal trends. Importantly, the inclusion of complication data adds to the novelty of this work, revealing a potential link between psychological intervention and tangible clinical outcomes such as dermatitis risk reduction.

Nonetheless, limitations must be acknowledged. First, the retrospective nature of the study, despite the use of advanced statistical methods to adjust for measured confounders, precludes definitive causal inferences. Variables such as patient motivation or baseline psychological flexibility, which may influence both ACT participation and outcomes, were not captured. Second, although ACT protocols followed established guidelines, variability in therapist expertise or patient engagement may have introduced heterogeneity. Future studies should include standardized fidelity checks and dosage assessments. Third, the study was conducted in a single tertiary center with relatively high socioeconomic representation, potentially limiting generalizability to underserved or rural populations. Fourth, reliance on self-report measures introduces subjectivity, although validated instruments with strong psychometric properties were used. Fifth, while multiple imputation was employed for missing data, the assumption of missing-at-random may not fully hold, especially for psychological variables.

Clinical implications of these findings are notable. The integration of ACT into routine stoma care offers a structured and theoretically grounded approach to supporting patient adaptation during a critical period of post-surgical recovery. Unlike brief reassurance or crisis-based counseling, ACT targets underlying processes that sustain long-term adjustment, including value reorientation and acceptance of distress. This may explain the progressive nature of improvements observed here, particularly between the 3- and 6-month follow-ups. From a health system perspective, the observed reduction in stoma complications implies that psychological interventions may yield downstream cost savings by preventing avoidable adverse events.

Future research should include randomized controlled trials comparing ACT with other psychological therapies (e.g., cognitive-behavioral therapy or problem-solving therapy) to elucidate comparative effectiveness. Identifying subgroups most likely to benefit—based on baseline distress, coping styles, or acceptance levels could facilitate personalized intervention. Cost-effectiveness analyses will also be important to determine the value proposition of integrating ACT into routine stoma management.

## 5 Conclusion

This study provides promising evidence that combining ACT with standard stoma care can meaningfully enhance self-efficacy, resilience, and quality of life in patients with permanent colostomies following colorectal cancer surgery. These psychosocial benefits were also linked with fewer stoma-related complications, and the strong connections among these outcomes point to a shared therapeutic effect. Although larger prospective studies are needed to confirm these findings, the results highlight the value of incorporating structured psychological support into the routine, multidisciplinary care of ostomy patients.

## Data Availability

The raw data supporting the conclusions of this article will be made available by the authors, without undue reservation.
